# Effect of vermiculite feed additive on the chemical, mineral, and amino acid compositions of quail meat

**DOI:** 10.14202/vetworld.2023.2431-2439

**Published:** 2023-12-06

**Authors:** Gulbanu Apdraim, Nurzhan Sarsembayeva, Bozena Lozowicka

**Affiliations:** 1 Department of Veterinary Sanitary Examination and Hygiene, Faculty of Veterinary Science, Kazakh National Agrarian Research University, Almaty, Kazakhstan; 2 Food Safety Laboratory, Institute of Plant Protection-National Research Institute, Bialystok, Poland.

**Keywords:** diet, feed additive, quail meat, quality, vermiculite, veterinary and sanitary assessment

## Abstract

**Background and Aim:**

Poultry farming plays a significant role in providing the population with high-quality and cheap meat products. The development and success of this industry depend on the proper use of available feed. As a rule, feed additives should compensate for missing elements in livestock diets. This study investigated the effects of vermiculite feed additives on the chemical, amino acid, and mineral compositions of quail meat.

**Materials and Methods:**

Texas Quail meat breed birds were randomly divided into three groups (35 heads each). Group I served as the control group, in which the birds were fed a standard diet without any supplements. The diet of Group II was supplemented with 3% vermiculite feed additive, whereas Group III received 5% vermiculite. The experiment was conducted over 120 days.

**Results:**

Quails in the experimental groups contained less moisture, had more ash and protein, and were richer in calories in terms of energy value. In terms of mineral composition, the calcium concentrations were 9.9% and 16.5% higher in the in the Group I and II, respectively. Furthermore, the highest percentages of nonessential and essential amino acids were found in the 5% vermiculite group. In the meat of quails that received 5% vermiculite, cadmium and lead salt levels were 20%–25% lower than those in the control variant.

**Conclusion:**

The results of our analyses confirmed that meeting the mineral requirements of quails improves animal productivity and meat product quality. Vermiculite as a feed additive improved the chemical, mineral, and amino acid compositions of quail meat when it was added to up to 5% of the feed dry matter. These results will help to improve the feed base of the poultry industry in the Republic of Kazakhstan.

## Introduction

Due to its high levels of B vitamins, macro- and microelements, and the presence of hydrolytic enzymes, dietary quail meat significantly reduces the load on pancreatic function and is a very valuable food in the diet of people with diabetes mellitus [1–3]. The poultry industry generally strives to improve the production performance and growth rate of birds by making selective changes, sometimes to the detriment of final product quality [4, 5]. Many factors, such as mineral nutrition, can affect the quality of animal and poultry meat [6, 7]. The problem of achieving optimal mineral nutrition for animal husbandry and poultry farming can be solved using complete feed and various supplements. More attention should be paid to natural minerals with various beneficial properties [8].

Vermiculite is a highly promising natural mineral, suitable for use in agriculture. Vermiculite is a silt mineral produced by decomposition during the weathering of biotite, phlogopite, chlorites, and silicates rich in magnesium [9–11]. The typical composition of this mineral was (Mg, Fe, Al)_3_ (Al, Si)_4_ O_10_(OH)_2_ × 4H_2_O. Depending on the origin of the deposit, other elements such as K, Na, Ca, Ti, and Cr may be present in small quantities [12].

Vermiculite is a widely used additive in animal and poultry feed to improve growth and health indicators and to reduce toxic residues and production costs [13, 14]. Vermiculite improves the sanitary and morphological properties of animal and poultry meat [15, 16]. Tyurina *et al*. [17] described the influence of a vermiculate-based mineral feed additive on the physiological parameters of Ross 308 cross-broiler chickens. The addition of limestone, monocalcium phosphate, and vermiculite improved the nutrient digestibility of broiler chicken feed. Zhidik *et al*. [18] found that enriching the main diet (MD) of quails of the Pharaoh breed with expanded vermiculite increased the live weight of birds and the safety of livestock during the experiment. Furthermore, the hematological parameters of the birds in the experimental group were superior to those of the birds in the control group. Bondar *et al*. [19] investigated the effects of vermiculite and Gumivet - feed concentrate Premix (Chişinău, Moldova) biologically active preparations in the diet of quails and found that the poultry meat they obtained was high in organoleptic, physicochemical, and bacteriological indicators, as well as biological value and safety for consumption. Introducing vermiculite into the diet of geese at 5.0 g/kg live weight increased the number of red blood cells, hemoglobin, hemoglobin content in red blood cells, and total blood serum protein, indicating more intensive metabolism and higher live weight. Overall, vermiculite positively affected the hematological mineral status of the birds.

The main vermiculite deposits are found in the United States, South Africa, Russia, and other countries. Vermiculite deposits are also found in the Republic of Kazakhstan. The annual demand for vermiculite in Kazakhstan is approximately 10,000 tons, which is attributed to its numerous applications [20]. In recent decades, the quail industry has become a promising new direction in industrial poultry farming in Kazakhstan, which can help meet the extremely high demand for dietary products such as quail meat and eggs [21]. The growing interest in this industry in Kazakhstan is due to the delicate consistency and juiciness of quail meat. Quail meat is an important source of biologically active and biogenic essential elements [22].

However, information on the effects of vermiculite from Kazakhstan deposits as a dietary supplement on the quality of meat and poultry products is limited.

Therefore, this study evaluated the effects of different doses of vermiculite feed additives on the chemical, mineral, and amino acid composition of quail meat.

## Materials and Methods

### Ethical approval

This study was approved by the Bioethics Committee (decision No. 156/7 dated October 13, 2022) of the Kazakh National Agrarian Research University. The analyses comply with the Code of Professional Ethics of Veterinarians and the ethical principles of animal research established by the European Convention for the Protection of Vertebrates Used for Experimental and Other Scientific Purposes.

### Study period and location

The study was conducted from October 2022 to February 2023 at SalemQus Limited Liability Partnership (LLP) (Almaty region, Eskelinsky district). Laboratory studies of quail meat were conducted at the Food Safety Research Institute of the Almaty Technological University of Kazakhstan.

### Animals

This study evaluated the effects of vermiculite additives on quails of the Texas Quail meat breed. The birds were reared for 120 days. The quails were randomly divided into three groups, each with 35 heads of 7-week-old quails. The origin, living weight, and general condition of the birds were recorded. Each group contained 35 heads of 7-week-old quail. The average weight of chicks at the beginning of the experiment was 32 ± 1 g. All experiments were conducted in triplicate.

### Raising birds and experiment design

During the experiment, feeding, weight control, and raising of the quails corresponded to typical housing and raising conditions on a quail farm. Chicks were raised in isolated battery cages (0.90 m × 1.54 m) with a mesh floor. All cages were equipped with automatic feeding and nipple drinkers, and chicks were free to feed and drink *ad libitum*. The main feed mixture contained the same components. The only difference was that the mixtures intended for the experimental groups were supplemented with a vermiculite feed additive. The birds in Group I (control group) were maintained exclusively on the MD. Group II received vermiculite feed additive at 3.0% of the dry matter of MD, whereas Group III received 5% feed additive. The scheme of the bird-feeding procedure is presented in Table-1.

**Table-1:** The design of the experiment on quail feeding.

No.	Groups	Feeding conditions	Number of quails
I	Control	100% MD	35
II	Experimental	97% MD + 3% V	35
III	Experimental	95% MD + 5% V	35

MD=Main diet, V=Vermiculite

The MD consisted of corn, wheat, sunflower cake, barley, soy meal, fish meal, salt, baking soda, limestone, choline chloride, methionine, lysine, threonine, minerals, and vitamin premixes. The nutritional value of 100 g of compound feed was 270 kcal of metabolic energy, 17.03 g of crude protein, 3.82 g of crude fat, 4.97 g of crude fiber, and 12.48 g of crude ash, which corresponded to the dietary recommendations for quail. The basic diet was formulated based on the requirements of the farm’s nutritional recommendations.

Vermiculite used in this study was derived from the Kulantau deposit (Kazakhstan), grade M-150, and fraction 0.5–3.0 mm. It is characterized by a high Fe_2_O_3_ content (20.59%), SiO_2_ (17.8%), K_2_O (8.18%), Al_2_O_3_ (7.22%), MgO (6.4%), TiO_2_ (2.27%), CaO (1.79%), and FeO (0.56%) [23]. In the experimental groups, vermiculite used in this study was replaced with a mineral premix and limestone. The composition of the control and experimental diets is presented in Table-2.

**Table-2:** Diet composition for control and experimental quail groups.

Ingredients, %	Groups		

	I	II	III
Wheat	10.00	10.00	10.00
Fodder corn	46.00	46.00	46.00
Barley	14.00	14.00	14.00
Soy meal	8.50	8.50	8.50
Sunflower cake	6.50	6.50	6.50
Baking soda	1.00	1.00	1.00
Vermiculite	0.00	3.00	5.00
Fishmeal	5.00	5.00	5.00
Lysine	0.40	0.40	0.40
Methionine	0.40	0.40	0.40
Threonine	0.20	0.20	0.20
Salt	0.50	0.50	0.50
Mineral premix^1^	1.00	0.00	0.00
Vitamin premix^2^	1.00	1.00	1.00
Limestone	2.00	0.00	0.00
Choline chloride	3.50	3.50	1.50
General	100	100	100

1 Mineral premix composition (mg/kg): zinc: 50; copper: 12; iodine: 0.3; cobalt: 0.2; iron: 100; selenium: 0.1; manganese: 110.

2 Vitamin premix composition: vitamin A: 12,000 international units (IU); vitamin D_3_: 2500 IU; vitamin E: 30 IU; vitamin K_3_: 2 mg; thiamine: 2.25 mg; riboflavin: 7.5 mg; pyridoxine: 3.5 mg; cobalamin: 0.02 mg; niacin: 45 mg; D-pantothenic acid: 12.5 mg; biotin: 0.125 mg; folic acid: 1.5 mg.

Physicochemical, mineral, and amino acid compositions and residual amounts of heavy metals were evaluated by collecting meat samples at slaughter and anatomical cutting of poultry carcasses. Quail carcasses were stored in a refrigerator at 4°C for 24 h for maturation. The pectoral muscles of the birds were used in this study.

### Investigation of the chemical composition of quail meat

The total moisture content in the muscle tissue was determined by drying the sample in an SHS-80-01 SPU drying cabinet (Smolenskoye SKTB SPU, Smolensk, Russia). To do this, 5 g meat samples were placed in pre-prepared weighing bottles and placed in a drying cabinet at 105°C for 2 h. The bottles were then cooled to room temperature (18–20ºC) in a desiccator and weighed.

To determine the fat content, 5 g meat samples were weighed and dried in a Petri dish in a drying cabinet at 105°C for 1 h. The dried samples were transferred to a sleeve and placed in the extractor of the Soxhlet apparatus (Almaty, Kazakhstan), and extraction was performed for 7 h. After the extraction was completed, the solvent was distilled from the extraction flask, and the extraction flask containing fat was dried in a drying cabinet at 105°C to a constant weight.

Protein content was determined as described subsequently. Anhydrous potassium sulfate (15 g), copper sulfate (0.5 g), and meat sample (2 g) were placed in a Kjeldahl flask, to which 25 mL of sulfuric acid was added and mixed. The flask was positioned at a 40° angle relative to the vertical position of the heating device, and mineralization was initiated by vigorous boiling. After the contents of the flask were completely clarified, boiling was continued for 90 min. The flask was cooled to 40°C and 50 mL of distilled water was added. The mixture was stirred and cooled to 18–20ºC. The contents of the flask were subsequently distilled, and three drops of paraffin oil and 100 mL of sodium hydroxide solution were added. Distillation was completed after obtaining at least 150 mL of distillate. The contents of the flasks were titrated with a hydrochloric solution. The titration results were used to calculate the total nitrogen mass fraction and subsequent conversion into proteins.

The ash content was determined by heating the sample cup for 20 min in a muffle furnace at 550°C, cooling it in a desiccator at 18–20ºC), and weighing it to an accuracy of 0.1 mg. The caloric content of meat was determined using the Alexandrov formula [24]:

X=[C - (F + A)] × 4.1 + (F × 9.3)

X is the caloric content of meat, kcal/kg; C is the amount of dry matter, g; F is the amount of fat, g; A is the amount of ash, g.

### Determination of macro- and microelements in quail meat

Sodium, potassium, magnesium, and manganese contents were determined using a KFK-3 spectrophotometer (NV-Lab Company, Russia). The obtained minerals were placed in a vessel, which was then placed in a fume hood that was kept open for at least 12 h. Next, the minerals were poured into a 20-mL tube and degassed in an ultrasonic bath for 5 min. The degassed minerals were diluted with water. The background signal of the device was compensated for using background solutions. Double absorption measurements of the calibration solutions at various concentrations were performed to calibrate the device. Subsequently, the absorption of the sample solutions was measured.

A bowl with a sample was placed on an electric stove to determine the iron, calcium, and zinc contents, and charring was performed to prevent excessive smoking. At the end of the smoke emission, the bowl was placed in an electric furnace, which was previously adjusted to approximately 250°C. Mineralization was continued until gray ash was obtained. The ash was cooled at 18ºC–20ºC, and moistened drop-wise with a nitric acid solution until saturation.

To determine the phosphorous content, mineralization of a 5 g meat sample was performed in a muffle furnace. The resulting ash was dissolved in 10 mL of nitric acid. The crucible was covered with a watch glass, heated for 30 min in a boiling water bath, and then cooled. The liquid produced was quantitatively transferred into a 100 mL measuring flask. The volume was brought to the mark with water, mixed, and filtered through a filter paper. The optical density of the solution was measured using a spectrophotometer at a wavelength of 430 nm in a glass cell, relative to the control solution. All analyses were performed in triplicate, and the results are expressed as average values (mg)/100 g of the product.

### Determining the amino acid composition of quail meat

The amino acid composition of the meat was determined by measuring the mass fraction of amino acids by capillary electrophoresis using a Kapel capillary electrophoresis system (Lumex, Russia). Pectoral muscles of the birds were used in the experiments.

### Determining the heavy metal content of quail meat

Residual heavy metals in quail meat were determined using a KVANT-Z.ETA atomic absorption spectrometer (Kortek LLC, Russia). The sample was placed on an electric stove, and carefully charred for sample preparation to prevent excessive smoking. After smoke emission, the bowl was placed in an electric furnace that was previously adjusted to 250°C and the temperature was gradually increased to 450°C. Mineralization was performed for 10–25 h until gray ash was obtained. The bowl containing the ash was removed from the electric furnace and cooled at 18–20ºC). The ash was then moistened drop-wise with a nitric acid solution until saturation. The acid was evaporated in a water bath, and the obtained substance was placed in a drying cabinet at 140°C. After cooling, the bowl containing the sample was placed in a cooled electric furnace.

### Statistical analysis

Data were analyzed using Microsoft Excel (Microsoft Corp., Washington, USA). The reliability of the data obtained was evaluated using student’s t-test. Differences were considered statistically significant at p ≤ 0.05.

## Results

### Chemical composition of quail meat

The addition of 3.0% and 5.0% vermiculite to the quail diet significantly enhanced the chemical properties of quail meat. Differences were observed in the chemical composition of the quail meat between the control and experimental groups. The meat from control quails had higher moisture content and lower ash, fat, and protein content. The meat from experimental birds had a lower moisture content, whereas fat, protein, and calorie contents increased (Table-3).

**Table-3:** Chemical composition of quail meat of the control and experimental groups.

Indicators (g/100 g)	Group (n = 35)		

	I (control)	II	III
Protein	20.62 ± 2.13*	21.35 ± 2.52	23.16 ± 1.95*
Fat	3.42 ± 0.25	3.39 ± 0.16	3.43 ± 0.42
Ash	1.12 ± 0.02	1.49 ± 0.22*	1.68 ± 0.11
Moisture	71.85 ± 3.65*	70.63 ± 3.69*	69.81 ± 4.32*
Caloric content, kcal/kg	128.6 ± 3.8	131.9 ± 4.2	134.7 ± 3.9*

* p ≥ 0.05

In terms of protein and ash contents, meat from the control group was inferior to that from the experimental group. For example, the average protein content in Group III was 23.16 g/100 g of product (p ≤ 0.05), whereas that in the control group was 2.5% lower at 20.62 g/100 g. In the second group, the protein content was 0.7% higher than that in the control group, and 1.8% lower than that in the third group. Thus, optimal indicators were achieved using 5% vermiculite.

The average ash content in the control group was 1.12 g/100 g, 1.49 g/100 g in the second group, and 1.68 g/100 g in the third group (p ≤ 0.05). Ash content was higher in the third group than in the control and second groups. However, even a smaller dose (3%) of vermiculite produced an ash content 0.37 g/100 g higher than that in the control group.

The fat content was comparable across the three groups, ranging from 3.39 to 3.43 g/100 g. The addition of vermiculite to quail diet led to a slight decrease in the moisture content of the meat. For example, the amount of moisture in the control group averaged 71.85 g/100 g, whereas it was 70.63 g/100 g in the second group, and 69.81 g/100 g in the third group. Measurements of moisture content and caloric content of the experimental groups were 1.6% and 2.8% lower than those of the control group, respectively. Furthermore, the caloric content of meat from the experimental groups was 3.3–6.1 kcal/kg higher than that of the control group (p ≤ 0.05).

The ash content was comparatively higher in the meat from both vermiculite treatment groups. The best meat quality indicators were achieved using 5% exfoliated vermiculite. These findings indicate that this dose completely fulfilled the mineral requirements of quails.

### Mineral composition of quail meat

The introduction of vermiculite increased the total mineral content of the quail meat. The mineral composition of the quail meat is shown in Table-4.

**Table-4:** The content of macro and microelements in quail meat.

Name of indicators, mg/100 g	Groups (n = 35)		

	I (control)	II	III
Macronutrients, mg/100 g			
Ca	13.6 ± 2.1	14.5 ± 1.1*	16.3 ± 2.4*
K	237.1 ± 5.6	239.2 ± 4.5	252.1 ± 3.2
Mg	24.8 ± 2.4	24.9 ± 2.2	25.1 ± 1.5
Na	51.8 ± 1.4	52.4 ± 1.6	52.8 ± 1.3
P	3.07 ± 3.5	3.25 ± 1.3*	3.41 ± 2.6
Microelements, mg/100 g			
Fe	4.51 ± 1.6	5.26 ± 0.5*	6.89 ± 2.4*
Mn	0.019 ± 0.001*	0.019 ± 0.001	0.025 ± 0.002
Zn	2.7 ± 0.6	2.8 ± 1.1	2.8 ± 0.8

* р ≥ 0.05

Analysis of the data on the mineral composition of meat revealed a general increase in the mineral content of meat in the experimental groups. In Group II, calcium and phosphorus contents increased by 6.2% and 5.4% (p ≤ 0.05), respectively. In Group III, the increases were more pronounced. An increase in calcium and phosphorus levels increases the biological value of meat because calcium is involved in regulating the porosity of the vascular endothelium, creating the structure of bone tissue and blood clotting.

The iron and manganese content in meat from the experimental groups indicated that iron levels were higher in the experimental groups. In Group II, the iron index was higher than that of the control group by 0.75 mg/kg, whereas that in Group III was higher by 2.38 mg/kg (p ≤ 0.05). This is because in these birds, under the influence of a natural mineral (vermiculite), hematopoietic processes proceeded most intensively with enhanced levels of iron, which is essential for such processes. In the muscle tissue, the manganese content in Group II was on average 0.019 mg/kg, which was comparable to that in the control group. In Group III, the level was higher than that in the control group by 0.006 mg/kg (24%). Furthermore, the potassium and sodium contents showed a tendency to increase in the experimental groups. Specifically, the potassium content in the control, second, and third groups was 237.1 ± 5.6, 239.2 ± 4.5, and 252.1 ± 3.2 mg/kg, respectively.

The magnesium and zinc contents did not significantly differ between the experimental and control groups. However, the highest concentrations of mineral elements were observed in Group III.

Thus, the use of vermiculite promoted mineral metabolism in quails. An increase in the intensity of metabolic processes improved the biochemical parameters of the muscle tissue. Thus, vermiculite enhanced metabolism, protective physiological mechanisms, and assimilation of basic nutrients, ultimately increasing the quality of poultry meat.

### Amino acid composition of quail meat

**Figure-1:**
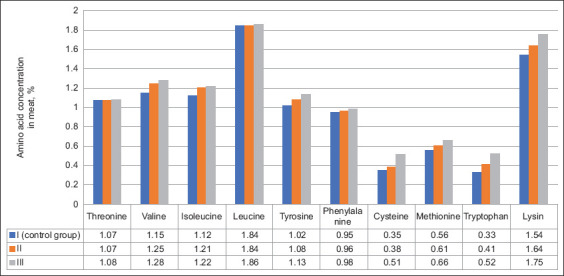
The content of essential amino acids in the muscle tissue of quails of the control and experimental groups (%).

**Figure-2:**
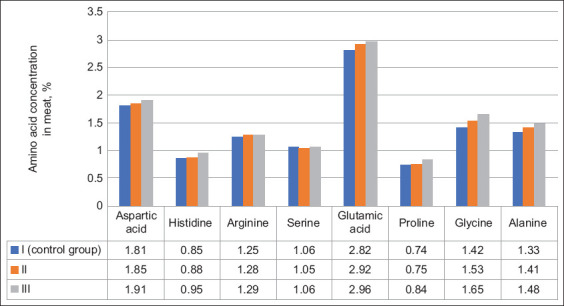
The content of nonessential amino acids in the muscle tissue of quails of the control and experimental groups (%).

Analysis of amino acid composition revealed differences in meat from the control and experimental groups (Figures-1 and 2). The amino acid composition of poultry meat in the experimental groups was characterized by relatively high levels of isoleucine, tyrosine, tryptophan, glutamic acid, proline, and glycine.

Furthermore, the total number of essential and nonessential amino acids was higher in the experimental groups than in the control group. Group III showed the highest essential amino acid levels per 100 g of absolute dry matter at 10.99% ± 0.16%. This was 1.06% higher than that in the control group (p ≤ 0.05).

The highest total nonessential amino acid levels were also observed in the third group of birds (12.98% ± 0.16%). In the control group, this indicator was 11.28% ± 0.18%, which was 1.7% lower than that in the third group (p ≤ 0.05). The number of nonessential amino acids in the second group was 11.67% ± 0.13%. The total amino acid content in the control group averaged 21.21% ± 0.35%, which was 0.91% and 2.76% lower than those in the second and third groups, respectively. Thus, feeding quails compound feeds containing vermiculite can significantly replenish deficiencies in the amino acid composition of poultry meat.

### Investigation of heavy metals in quail meat

The cadmium and lead levels in the control and experimental groups did not exceed the maximum permissible concentration (MPC) (Table-5). The average cadmium content in the muscle of birds from the control group was 0.0008 mg/kg, whereas it was 0.0006 mg/kg in the second and third groups. With the addition of vermiculite to the diet, a significant decrease (p ≤ 0.05) in cadmium was noted at 21.3%. Furthermore, the average lead content in the second and third groups was significantly (p ≤ 0.05) lower than that in the control group by 0.0002 and 0.0003 mg/kg, respectively. The MPC of lead in meat is 0.5 mg/kg. Thus, even the smallest dose of vermiculite (3%) significantly improved the sorption properties of lead. The lead content in meat from the control group did not exceed that of MPC (0.0012 mg/kg). Furthermore, arsenic and mercury were not detected in meat samples from the control and experimental groups.

**Table-5:** The content of heavy metals in quail meat when using the vermiculite feed additive.

Name of indicators, mg/kg	Received (n = 35)			MPC

	I (control)	II	III	
*Hg*	Not found	Not found	Not found	0.03
*As*	Not found	Not found	Not found	0.1
*Cd*	0.0008 ± 0.00002	0.0006 ± 0.00002*	0.0006 ± 0.00001*	0.05
*Pb*	0.0012 ± 0.0001	0.0010 ± 0.0001	0.0009 ± 0.0001*	0.5

MPC=Maximum permissible concentration

Table-5 shows that in the meat of quails that received 5% vermiculite, cadmium and lead salt levels were lower than those in the control by 20%–25%. Thus, the data indicate that supplementing feed with a natural mineral (vermiculite) reduces the heavy metal content in quail meat.

## Discussion

This study identified vermiculite as a promising new additive to poultry feed that increases the productivity of birds and improves the quality of poultry products. The chemical composition of poultry meat is not constant and depends significantly on the type, physiological condition, age, sex, habitat, and type of feed and cultivation [25]. Banaszak *et al*. [26] studied the effects of natural minerals on the quality of broiler meat and found that the average protein content in the muscles of birds was significantly higher when natural minerals were added to the MD.

The introduction of vermiculite into quail feed improved the digestibility of nutrients and optimized the metabolic process in the quail. Bondar *et al*. [19] noted that exfoliated (loose porous material) vermiculite in poultry feed increased resistance to diseases and efficiency of feed consumption, even improving the appetite of chicks. Tyurina *et al*. [27] found that a mineral supplement containing vermiculite improved the digestibility of feed nutrients and the biological value of broiler chicken meat.

Our findings on the ash and moisture content of quail meat revealed that vermiculite increased these quality indicators. The highest ash content was observed in the birds that received 5% vermiculite (1.68 g/100 g). Analysis of the chemical composition of the meat samples from the control group showed a higher moisture content than those from the second and third groups by 1.7% and 2.8%, respectively. Similar results were reported by Banaszak *et al*. [28], who found the lowest moisture content in the breast meat of animals that received aluminosilicate as a feed additive. In this study, the caloric content of meat from the control group was 3.5% lower than that of the experimental groups, indicating lower protein levels.

Microelements (potassium, sulfur, phosphorus, sodium, chlorine, and calcium) and macroelements (iron, zinc, copper, and fluorine) are important in poultry meat [29]. The addition of vermiculite increased calcium levels by an average of 11.6% and phosphorus levels by 7.8% compared with those of the control group. These results confirmed those of Razanova [30], who also added mineral additives to the MD of quails and observed an increase of 37.7%–40.5% in calcium content and 8.9%–17.4% in phosphorus content.

Potassium controls the water/salt balance in the body of birds, whereas manganese promotes oxidative processes and participates in fat metabolism [31]. The potassium and manganese concentrations in muscle samples from Groups I and II were 237.1–239.2 and 0.019 mg, respectively. However, the concentrations of potassium and manganese in Group III were the highest (252.1 and 0.025 mg), with a difference of 5.5% and 24%, respectively, compared to those in the control group.

Mineral supplementation of quail feed also enhanced the iron content of quail meat. The iron content in meat from the experimental groups was higher than that of the control group, with the highest concentration found in meat from Group III (6.89 mg), which was 2.38 mg higher than that from the control group (4.51 mg). These data show that the mineral elements that constitute vermiculite are vital to quail physiology. Iron is involved in hemoglobin synthesis and the redox process, whereas zinc is involved in bone tissue formation, nucleic acid metabolism, protein synthesis, and eggshell formation [32].

After analyzing the indicators of the mineral composition of quail meat, we concluded that the total mineral content of the meat from the experimental groups was higher. The increase in the total amount of macro and microelements in the meat of the experimental groups may be associated with the high mineral content of vermiculite.

Quail meat is considered an excellent food for human consumption because of its protein content, specifically the types and amounts of essential amino acids the meat contains [33]. Our analysis of the amino acid content of quail meat revealed that the addition of 5% vermiculite to the MD of birds significantly increased essential amino acid levels by 9.6% and nonessential amino acid levels by 13%. However, the total amino acid content reported in our study was lower than that reported by Genchev *et al*. [34]. They obtained 22.21 g of amino acid concentrations from the breast meat of 42-day-old quails. A likely explanation for this difference may be the age of the quails at the time of slaughter. The increased nonessential and essential amino acid contents in muscle tissue contribute to the activation of enzyme systems, namely, the synthesis of peptide and protein hormones, and an increase in protein-synthesizing liver functions, which further accompanies an increase in blood protein concentration, normalization of the colloidal osmotic pressure of tissues, and water–salt metabolism. A similar increase in amino acid levels in poultry and animal meat has been reported in several studies. When agrominerals are introduced into the diet of animals, the abrasive effect of minerals in the intestines induces changes in the crypt villi system, increasing the surface area for the nutrient absorption and feed conversion. This ultimately increases the protein content of meat [35]. Consequently, using vermiculite as a feed additive in quail feed increases the biological value of the poultry products obtained.

Cadmium enters the bodies of birds and animals mainly through food. The MPC of cadmium in meat is 0.05 mg/kg. In the meat from the control group, the average cadmium content was 0.0008 mg/kg. This amount was significantly decreased in the meat of the experimental groups at 0.0002 mg/kg.

Lead can accumulate in many organs and tissues. The lead content in meat from the first group was 0.0012 mg/kg, whereas it was 0.0010 and 0.0009 mg/kg in the second and third groups, respectively. These differences in lead content imply the excellent adsorption and ion-exchange properties of vermiculite. Darin and Kerdyashov [36] described the sorbing properties of natural aluminosilicates, including vermiculite, in detail. Thus, enriching quail feed with 5% vermiculite reduces the lead and cadmium levels in poultry meat.

## Conclusion

These findings confirm that vermiculite as a feed additive enhanced the chemical, mineral, and amino acid composition of meat obtained from quails within physiological norms. Furthermore, vermiculite significantly reduced the amount of heavy metals in meat. The results show that the high mineral nutritional value of vermiculite added to standard feed promotes the production of high-quality poultry meat products. These findings are valuable to the poultry industry, in developing countries, particularly in the interest of enhancing the properties and quality of poultry meat and improving the health and safety of consumers. As demonstrated herein, vermiculite can be used as an additive in quail feed at 5% of the dry matter of the standard diet.

## Authors’ Contributions

GA and NS: Conceptualization, design, and planning of the study, data collection and analysis, and critical review of the manuscript. NS, BL, and GA: Conducted the research, statistical analysis, and drafted the manuscript. BL, TA and NS: Sampling and delivery of samples and conducted the study. All authors have read, reviewed, and approved the final manuscript.
